# The Experience and Impact of Digital Technologies on Indigenous Populations in New Zealand During the COVID-19 Pandemic and Cyclone Gabrielle: The Kaupapa Māori Methodology

**DOI:** 10.2196/73974

**Published:** 2025-10-28

**Authors:** Dianne Wepa, Shiji Thomas, Md Shafiqur Rahman Jabin

**Affiliations:** 1 School of Community and Public Health, Auckland University of Technology, Auckland, New Zealand; 2 Faculty of Health Studies, University of Bradford, Bradford, United Kingdom; 3 School of Health and Society, University of Salford, Salford, United Kingdom; 4 Department of Medicine and Optometry, Linnaeus University, Universitetsplatsen 1, Kalmar, 392 31, Sweden, 44 7915 673 612

**Keywords:** community service, COVID-19, Cyclone Gabrielle, digital exclusion, digital solutions, disaster management, health inequalities, Māori kaumātua, Ngāti Kahungunu, training and education

## Abstract

**Background:**

Pandemics, such as COVID-19, and climate change–related catastrophic weather events are increasing, impacting social connectedness within communities by disrupting social cohesion, increasing loneliness, and affecting mental health and social well-being. Digital technology, in addition to being used for communication, education, and business transactions, also plays a vital role in maintaining a country’s health and well-being, as well as sustaining economic growth.

**Objective:**

This study aimed to explore the experiences of Māori kaumātua in using digital technology to meet their health needs within Ngāti Kahungunu, North Island, New Zealand, during the COVID-19 pandemic and Cyclone Gabrielle.

**Methods:**

This qualitative study employed the Kaupapa Māori methodology to understand the challenges, resilience, and approaches used by Māori to maintain connectedness and access essential services. An inductive approach to thematic analysis, as recommended by Braun and Clarke, was used to ensure a thorough and robust data analysis. The user characteristic was assessed on a semantic level using the information provided in the narrative text.

**Results:**

The findings highlight the role of digital technology in disaster management and underscore the urgent need to address digital disparities in support of vulnerable populations. In this study, 14 individuals were interviewed, comprising 71% (n=10) women and 29% (n=4) men. These participants fell into different age groups, with 9 participants being 65 years or older (older adults). Of the total participants, 43% (n=6) were limited users, 43% (n=6) comprised confident users, and the rest (n=2; 14%) were normal users. A total of 6 themes emerged from the interview data: social connectedness and resilience, digital literacy and access to information, barriers to telecommunications and digital technology, cultural appropriateness and psychological barriers, perceived threats of feeling insecure, and impact on mental health and emotional well-being.

**Conclusions:**

Vulnerable situations such as pandemics and extreme weather events can have tremendous effects on the lives of Indigenous people who live remotely. The study also focused on the actions that should be taken to mitigate these challenges and overcome difficult circumstances, such as the pandemic and the cyclone. The recommendations include a better health care system and improved coordination among care providers, user-friendly digital solutions, ensuring local funding and community services, establishing training processes for basic digital skills, and fostering leadership and partnerships with Indigenous New Zealanders.

## Introduction

### Background

Pandemics, such as COVID-19, and climate change–related catastrophic weather events are increasing, impacting social connectedness within communities by disrupting social cohesion, increasing loneliness, and affecting mental health and social well-being [1,2]. Displaced populations and resource scarcity can reduce social functioning. However, the breakdown of local support systems is one of the key factors that maintain social functioning and can have a significant impact on communities. Psychological impacts such as anxiety and fear of future events diminish social engagement, particularly for vulnerable groups such as older people [1,2]. Indigenous older people are generally more vulnerable (or at risk) in each of those domains, such as COVID-19, natural disasters such as Cyclone Gabrielle, and digital technologies [3-5].

### The Context of the COVID-19 Pandemic in New Zealand

New Zealand, a small island nation with a population of approximately 5 million, is a diverse community, with significant representation from the Māori communities (16%), Asia (15%), and the Pacific Islands (7%), as well as Europe and other countries (62%) [6]. Māori are the Indigenous people of New Zealand. They migrated from Polynesia to New Zealand more than 800 years ago. Their tradition and culture are deeply rooted in respect for nature, community, and their kaumatua (elders) [7]. The New Zealand government introduced new strategies in response to the COVID-19 pandemic. One of the strategies includes a 4-tiered alert system that indicates the severity of the COVID-19 situation and corresponding measures [8]. This system, which ranges from Alert Level 1 (prepare) to Alert Level 4 (lockdown), was not only highly regarded worldwide but also proved effective in managing the crisis [9].

Most of the workforce, excluding essential workers such as staff from supermarkets, hospitals, and transport, was advised to work remotely during the lockdown period [9]. Daily media briefings, led by government officials like the Prime Minister and Director-General of Health, were broadcast on the radio to keep the population updated about the impact of the disease in the country and the plans to eliminate it [10]. Websites and the national emergency broadcast system were used to advise the public regarding safety measures [11]. Important information from the “Unite Against COVID-19 website” and the number for free helplines were also shared on social media platforms, such as Facebook, Twitter, Instagram, LinkedIn, and WhatsApp, by government agencies [4]. These ways of communication and working meant that people had to rely on digital technology during the crisis period.

### The Context of Cyclone Gabrielle in New Zealand

Cataclysmic weather episodes are increasing globally, adversely contributing to the rise in health-related risks [12], food security, livelihoods, clean water supply, and human security [13]. Climate change–related health effects are variable for different populations as they depend on geographical positioning, health system capacity and capability, demographics, and the disease burden of communicable and non-communicable diseases [14]. The world faces a distinctive challenge with the rapidly changing climate and the rising frequency of extreme weather events. In the recent decade, the prevalence of extreme weather events has escalated due to climate change and global warming [12,14]. Coronese and colleagues established a compelling relationship between climate change and the heightened frequency and severity of extreme weather events in numerous regions of the globe [15]. The Intergovernmental Panel on Climate Change [16] delineates the implications of climate change and climate-induced extreme weather events on socioeconomic systems, including crop damage, heightened wildfire risk, more frequent power outages, increased susceptibility to food or water shortages, and political system vulnerability [5].

Within the context of New Zealand, severe weather and climate events are frequently experienced, primarily associated with flooding that disconnects different communities and groups of people from accessing support in the form of health care services, medical and food supplies, safe drinking water supply, and safe areas for rehabilitation during disaster occurrences [17]. The year 2023 began with a series of emergencies declared in Aotearoa, New Zealand [18]. Regions, such as Coromandel/Thames and Te Tairawhiti Bay of Plenty, were hit by Cyclone Hale [19,20], not only causing considerable damage to the livelihoods of the residents but also uprooting the entire coastal ecosystem from its essence [17]. Disastrously, this was followed by Cyclone Gabrielle, which caused significant flooding in many different parts of Tamaki Makaurau and caused the government to declare a national emergency [21,22]. The devastating impact of Cyclone Gabrielle was particularly evident in regions of Hawke’s Bay and Gisborne [21]. The floodwater left thousands stranded and trapped in high-risk landslide areas [23].

### The Use of Digital Technologies by Indigenous New Zealanders

Over the last 2 decades, significant use of technology has been seen, mainly for communication, education, and business purposes [24]. However, in 2019, the Department of Internal Affairs reported that 1 in 5 of the population, mainly people from Māori and Pacific Islands, people living in social housing or rural areas, older persons, and people self-identifying as the unemployed and the disabled, did not have access to or the skills to use digital technology [25]. Digital technology in this context refers to electronic devices, such as computers and mobile phones, and their infrastructure (connection modalities) used for communication and sharing information. The report also highlighted that digital exclusion affected their access to education and communication and significantly impacted a person’s well-being, underscoring the urgent need to address this issue, and different recommendations were proposed to overcome digital exclusion [25].

Similarly, many studies have reported health inequalities with Māori, resulting in high morbidity and mortality rates [26,27]. The incidence rate of complications and deaths has been high in the Māori communities during previous outbreaks of influenza and other communicable diseases [28]. However, there is potential for introducing positive changes by understanding the lived experiences of Māori people in using digital technology to meet their health care needs.

### Study Aims

The primary aim of this exploratory qualitative study was to understand the lived experience of Māori communities in using digital technologies to meet their health care needs during the simultaneous occurrence of the COVID-19 pandemic and Cyclone Gabrielle. This study focused on the experience of Māori communities, particularly the older generation (known as kaumātua), during the COVID-19 pandemic and Cyclone Gabrielle, emphasizing the importance of digital technology in maintaining social connectedness and accessing vital services. The study explored the following research questions:

1. What was the experience of day-to-day life using digital technologies for the Indigenous New Zealanders during the COVID-19 pandemic and Cyclone Gabrielle?
2. How did the pandemic and the natural disaster affect the use of digital technologies and access to information?
3. What recommendations should be considered for using digital technologies and information access during a pandemic and natural disaster?

## Methods

### Overview

This was an exploratory qualitative study based on the Kaupapa Māori methodology [29]. This methodology involves research developed, conducted, and reported by Māori to promote and support their community. This research approach is grounded in Māori worldviews, values, and practices. It is a decolonizing methodology developed by and for Māori, seeking to challenge Western-dominated ways of knowing and conducting research. The Kaupapa Māori methodology prioritizes Māori aspirations, language, culture, and autonomy throughout the research process [29-31]. Kaupapa Māori research is often participatory and collaborative, focusing on creating knowledge in partnership with Māori communities. In this sense, it aligns strongly with the interpretivist approach, as it emphasizes the importance of understanding people’s subjective experiences, values, and cultural contexts [32].

### Data Collection

In this study, 14 Māori families from various communities within the tribe or iwi, known as Ngāti Kahungunu, participated in semistructured interviews conducted between December 2022 and February 2023. A purposive sampling method and the interpretivist approach were employed to select participants from Māori communities, as this Phase 2 study builds upon a previous research conducted by Wepa and colleagues [30], which involved interviewing a small population group. The population group represented the senior members and older adults of Māori families since they are generally more vulnerable (or at risk) in each of those domains, such as COVID-19, natural disasters such as Cyclone Gabrielle, and digital technologies. Participants who were at increased risk for adverse events were excluded. Participants ranged in age from 45 to 90 years old. A participant information sheet was provided to all participants.

These families were interviewed as a group as per the Kaupapa Māori methodology. Under this methodology, research was developed, conducted, and reported by Māori to promote and support their community [29-31]. A research assistant connected to each community supported the primary investigator in conducting the semistructured interviews from December 10, 2022, to February 16, 2023. The interviews lasted an average of 40 minutes, with the longest interview being approximately 90 minutes. The interviews were targeted at the family’s senior member, such as older adults; in some cases, other family members, such as daughters, sons, and grandchildren, participated in a support role. This is because some older adults experienced difficulties with hearing or vision, making it challenging for them to participate in an interview. In such cases, family members helped by clarifying questions and reading materials aloud. Semistructured interviews were conducted by JTH and RTH (research assistants) using the interview guide (Textbox 1). The research assistants were chosen since they were familiar with the values and cultural background of the Indigenous community in New Zealand. However, the interview was not only restricted to the interview guide but also included additional social demographic information, such as age, gender, location, living conditions, which were also collected. The interviews were audio-recorded and supplemented by written field notes.

Textbox 1. **Interview guide for semistructured interviews.**

**Interview guide**

1. How did you use digital technology (during COVID-19 and Cyclone Gabrielle)?
2. Given your experiences, do you have any suggestions for what improvements could be made?
3. What process do you think providers of services could consider?
4. What process do you think (whanau, hapu, iwi) people could consider?
5. Is there something else you would like to share?
6. Is there anything else you would like to ask me?

### Data Analysis

The recordings were transcribed to free-text narratives by RS (research assistant), as the researcher’s background, training, and familiarity with the Indigenous community aligned with the subject matter. The free-text narratives were analyzed by MSRJ and DW and reviewed by ST to minimize bias and enhance the validity of the results, since these researchers’ expertise lies within the realm of qualitative thematic analysis. An inductive approach to thematic analysis, as recommended by Braun and Clarke, was used to ensure a thorough and robust data analysis. This comprehensive approach was chosen to ensure the validity and reliability of the results, instilling confidence in the research findings [33], which involved identifying keywords and phrases from the transcripts to indicate potential relevant concepts. The extracted concepts were then grouped into a number of themes of similar nature. Discussions were held between MSRJ, ST, and DW to achieve a consensus when there was a difference in opinion, ensuring a collaborative and inclusive research process [34,35]. This collaborative approach was chosen to ensure that all perspectives were considered, making the research process more inclusive and comprehensive [34].

### Ethical Considerations

Written informed consent was obtained from them before the interviews. The process of obtaining consent involved explaining the study, its potential risks and benefits, and the participants’ rights. Participation in the study was voluntary, and the participants were given the option to attend interviews at their homes or a meeting place of their choice. Transportation was arranged for participants who attended interviews at a communal place. The research grant covered participant travel costs. Ethics approval was granted by the Auckland University of Technology Ethics Committee on 10 November 2022 (Ref No. 22/30). Any personal or sensitive information collected was deidentified, and the free-text narratives were aggregated for analysis.

## Results

### Participants Characteristics

For this study, 14 individuals were interviewed, comprising 71% (n=10) women and 29% (n=4) men. These participants fell into different age groups, with 9 participants being 65 years or older (older adults; Table 1).

Of 14 participants, 6 lived in a rural community, with 1 residing outside of it; 2 close to it; 2 in central Hastings, Hawkes Bay; and 1 at regional Ngati Kahungunu area. Among the remaining participants, 3 resided in Havelock North, 1 in Matahiwi Marae Base, 1 in regional Ngati Kahungunu area, 1 at the central location, and 1 outside Hastings, Hawkes Bay (Table 1). These are the places where Māori populations were mainly represented.

The participants older than 65 years and living with disabilities or preexisting health conditions were particularly vulnerable. The health conditions ranged from mobility, vision, hearing, cardiovascular or respiratory diseases, reliance on medication or health services for their daily needs and overall well-being.

All participants were isolated at home during the COVID-19 pandemic, with 64% (n=9) living with family members or dependents, such as sons, daughters, and grandchildren, and the rest (n=5; 36%) isolated alone with no family members. Of all, 36% (n=5) of the total participants, who were isolated alone, were dependent on others, such as friends, family (not living with them), and community members, for support (Table 1). Participants reported strong kinship networks as a substantial social capital central to the communities, enabling them to practice strong social connectedness or cohesion.

All participants except 1 received help from the local community (eg, Whānau, Marae) members, family members, or local health providers (eg, Māori; Table 1). They received food, water, sanitizing products, and other health needs. Some individuals were provided with services, such as pick-ups and drop-offs for health appointments and vaccinations, by local health providers. They could rely on community members and health providers for home visits, funding, access to COVID-19 information, and other medical needs.

Some participants felt comfortable and confident offering support and advice to other community members affected by COVID-19, as well as distributing food packages, sanitizing products, and medical kits. Other participants received little help during the COVID-19 pandemic; instead, they relied on non-family members for support and struggled to find information independently (Table 1). The person who did not receive support during the pandemic experienced worsening unmet needs, reduced access to care, increased distress, and heightened loneliness.

Of the total participants, 50% (n=7) had a good or excellent understanding of COVID-19 information and requirements (eg, isolation, staying home; Table 1). Of these 7 participants, 5 were either health workers, former health workers, or affiliated with health organizations within the community. This group of participants was proactive in offering health advice, including guidance on accessing information and selecting suitable digital media. Their support extended beyond the domain of information and technology, including vaccinations through local health providers and transportation for elderly individuals who were ill. The remaining 50% (n=7) lacked a good understanding (fair or basic) of COVID-19, needing help and heavily relying on other family members for information and health support (Table 1).

**Table 1.** Participant characteristics.

P^a^	Age (years)	G^b^	Living area	Living with FM^c^ or dependents or alone	Help received	Understanding of COVID-19 (health care workers)	DT^d^ usage	Internet access
P1	55+	M^e^	Mathiwi Marae Base	FM or dependents	Yes	Good (Ministry of Health)	Confident	No issues
P2	55+	F^f^	Paki Paki, Hastings Hawkes Bay	FM or dependents	Yes	Fair	Normal	No issues
P3	85+	M	Close to central Hastings Hawkes Bay	Alone	Yes	Basic	Limited	Had issues
P4	55+	F	Havelock North	FM or dependents	Yes	Excellent (midwife)	Confident	No issues
P5	60+	F	Paki Paki, outside Hastings Hawkes Bay	Alone	Yes	Good (volunteer)	Confident	Had issues
P6	65+	M	Flaxmere area	Alone	Yes	Basic	Limited	Had issues
P7	40+	F	Paki Paki, close to Hastings Bay	FM or dependents	Yes	Good (caretaker)	Confident	Had issues
P8	65+	F	Havelock North	Alone	No	Fair	Limited	Had issues
P9	65+	F	Central Hawkes Bay	FM or dependents	Yes	Good (ex-health care worker)	Confident	Had issues
P10	85+	F	Regional Ngati Kahungunu area	FM or dependents	Yes	Fair	Limited	No issues
P11	65+	F	Outside Hastings	FM or dependents	Yes	Good	Normal	Had issues
P12	85+	M	Havelock North	Alone	Yes	Good	Confident	Had issues
P13	75+	F	Central Hawkes Bay	FM or dependents	Yes	Fair	Limited	No issues
P14	65+	F	regional Ngati Kahungunu area	FM or dependents	Yes	Basic	Limited	Had issues

a P: participants.

b G: gender.

c FM: family member.

d DT: digital technology.

e M: male.

f F: female.

### Sources of Information

A number of sources of information were identified: the Ministry of Health website, COVID number (eg, 0800), TV and radio news or updates, COVID app (eg, COVID Sign in), tracking app with QR code, and social media (eg, Facebook). To access information and health requirements, the participants used their own devices, including cellular phones, smartphones, telephones, TVs, computers, and laptops.

A total of 5 (36%) of the participants reported having access to the internet with no issues, while the rest (64%) described their internet access as inconsistent and expensive (Table 1).

### Digital Technology User Characteristics

Of the total participants, 43% (n=6) were limited users, 43% (n=6) comprised confident users, and the rest (n=2; 14%) were normal users (Table 1). The user characteristic was assessed by using the information provided in the narrative text on the semantic level (without making any assumptions).

Before the pandemic and the cyclone, the limited users did not know how to use such technologies. Therefore, during the pandemic and the cyclone, these users had to depend on family members, such as daughters and grandchildren, to access government information or regular updates from the TV. The grandchildren indicated that the use of those technologies remained limited or did not improve among the senior family members after the pandemic and the cyclone.

*I had to rely on my granddaughter, who could access information through Facebook.*

[P13]

Confident users could navigate and access various information sources and the online world and regulate social media (eg, Facebook), frequently using different apps before the pandemic and the cyclone. The frequent use of digital technologies enabled these participants to quickly search for COVID-19 information during the pandemic. They could use a QR-scanning app to access information on health requirements and track the latest emergency updates from the government (eg, the Ministry of Health website). The increased use of technology during the pandemic and the cyclone also enabled the participants to connect with family members through social media. After the pandemic and the cyclone, confident users continued to use technologies more efficiently and regularly. They upgraded their knowledge and use of various other apps, such as Microsoft Teams (virtual meetings), and helped other family members with shared information.

*Navigating information through various means made me feel confident and act on necessary actions.*

[P12]

Before COVID-19 and the cyclone, most people used digital technology mainly for social media, such as Facebook. Their regular use of social media helped them keep in touch with their family, and apps such as “COVID Sign-in” supported them in accessing updated information from the government about the ongoing pandemic and the cyclone. Although this user group continued to use various social media and apps to a significant extent, they relied on their family members for extended usage of apps, such as QR scanning, after the pandemic.

*I use Facebook regularly. This helped me accessing updated information from the government.*

]P2]

### Themes That Emerged as a Result of the Study

In total, 6 themes emerged from the interview data: social connectedness and resilience, digital literacy and access to information, barriers to telecommunications and digital technology; cultural appropriateness and psychological barriers, perceived threats of feeling insecure, and impact on mental health and emotional well-being.

#### Theme 1: Social Connectedness and Resilience

Kaumātua, with their resilience and adaptability, have been the cornerstone of fostering community resilience. Their effective use of digital platforms to stay connected with whānau and access health services has been a testament to their resourcefulness. Their collective action, community solidarity, and reliance on traditional support networks have been instrumental in overcoming the challenges posed by isolation during COVID-19 and Cyclone Gabrielle [3]. Most participants felt safe and fortunate to have family and friends to rely on during the COVID-19 pandemic. The importance of love and a sense of belonging within family and community was expressed by many participants, while experiencing social isolation during the pandemic and cyclone. They felt confident in the information provided by family members, such as grandchildren, and assessed their health needs, including vaccination for COVID-19. They could also rely on the community for help, as they felt safer and more confident within it. They felt the need to connect with relatives living outside New Zealand. This further enabled them to rely on digital technology (eg, smartphones) to stay updated and informed, not only for themselves but also for their family, the community, and the outside world.

*A lot of good medicine is basic love, how do we support each other? Through aroha (love*).

[P4]

*No department knows me better than my moko (grandchild) does.*

[P10]

The participants considered connectedness as an essential factor in maintaining good health and well-being. As such, social connectedness can help neutralize vulnerabilities within a socioeconomic context through neighborhood networks, community cohesion, and trustworthiness, which can empower community members to work toward shared goals and objectives during disasters.

*You can only look to your community for help, nobody else.*

[*P10*]

#### Theme 2: Digital Literacy and Access to Information

The digital divide was evident, with many participants relying on the tech-savvy younger generation for technology use and access to information. This intergenerational support has been crucial in bridging the gap. However, some community individuals lacked the basic digital skills needed to navigate the internet effectively, and others were required to develop advanced digital skills to enhance their confidence and motivation. The impact of limited digital literacy on independent access to health-related information is significant, and coupled with inconsistent internet connectivity and high costs, it hinders their ability to access health-related information independently [3].

*First it was COVID-19 and now it’s the cyclone, no coordinated approach by the government–where will it end?*

[P6]

The participants relied heavily on social media to stay in touch with their family members. However, their lack of knowledge and access to digital devices hindered their ability to maintain communication during periods of isolation and the cyclone. Difficulty in using digital devices affected their ability to access online health facilities. They suggested introducing more user-friendly digital solutions to overcome these challenges. Moreover, local care providers demonstrated an apparent lack of coordination among themselves, which could have been further improved by exchanging and updating information promptly and delivering one-on-one services as needed.

*We used to have a health clinic, now you’re told to go online, nobody around to help do that if you don’t have someone in the family to help.*

[P3]

#### Theme 3: Barriers to Telecommunications and Digital Technology

During Cyclone Gabrielle, widespread power outages and telecommunications failures severely affected the community’s ability to communicate and access emergency services [3]. Participants emphasized the need for improved infrastructure, better internet connections, and localized communication channels to address these challenges.

*A rich man moved out to our beach and complained about the internet, then a new tower got built soon after.*

[P11]

*People are building empires, need to cut the red tape and put that money back into the people.*

[P9]

It was a challenging situation for those who were not digitally savvy, as they had to rely heavily on others for health needs and information. The government should have played a more significant role in addressing these gaps; for example, providing appropriate “match support” could have improved the situation.

*Good phone and internet coverage was only available with one company.*

[P7]

#### Theme 4: Cultural Appropriateness and Psychological Barriers

A preference for face-to-face interactions over digital communication was evident for the Māori communities. Various concerns about online safety and privacy, as well as the potential fear of losing the value of their own culture and language, contributed to the reluctance to engage with digital technologies. This necessitated digital inclusion initiatives and online services to be designed through the lens of the local community, incorporating their cultural values, learning styles, and needs. Fostering local community-led digital projects and ensuring online environments are safe and respectful is required. Ensuring online safety is crucial to building trust and confidence in the digital world. It is equally important to provide access to culturally relevant content, making users feel understood and catered to.

*If we are supposed to use QR codes, where is the privacy around that?*

[P1]

#### Theme 5: Perceived Threats of Feeling Insecure

The frequent visits of health providers were seen as “unwanted people” turning up at home, posing a significant risk to security during the time of isolation. Social media would have been a better choice than meeting in person, as it reduced the risk of contracting COVID-19 associated with in-person visits. For example, one of the participants felt intense fear and uncertainty when returning to her own community, as she was unsure about how it might affect her if she contacted her community members.

*She didn't like people or unwanted people turning up at her home (during Covid-19). She felt unsafe and insecure.*

[P5]

#### Theme 6: Impact on Mental Health and Emotional Well-Being

Following the cyclone, the area experienced constant power cuts and load shedding, which was particularly worrisome since the internet and phone lines were down for most of the time. However, in the face of these challenges, the Māori community came together in a display of unity and support. The disruption to community services and communication, combined with the displacement from homes and separation from family and community, particularly elders and children, loss of property, and disruption to cultural practices, had a significant impact on the mental health and well-being of Māori communities. But the community’s solidarity was a powerful force that helped overcome these difficulties.

*At some point, I felt mentally disturbed and did not know what to do.*

[P6]

## Discussion

### Principal Findings

This study aimed to explore the experiences of people from Māori communities in using digital technology to meet their personal health needs during the COVID-19 pandemic and Cyclone Gabrielle. It examined their ability to access digital devices and their skills and capabilities in using these devices to access vital information. Our study’s findings provide valuable insights into the role of digital technology in disaster management and underscore the urgent need to address digital disparities in support of vulnerable populations, such as older adults. In total, 6 themes emerged from the interview data: social connectedness and resilience, digital literacy and access to information, barriers to telecommunications and digital technology, cultural appropriateness and psychological barriers, perceived threats of feeling insecure, and impact on mental health and emotional well-being. This section presents the interpretation of the results and their significance by relating them to existing research, particularly in terms of the impact of digital technologies during the pandemic and the natural disaster, followed by a set of recommendations, and the strengths and limitations of the study.

Maintaining social connectedness during disasters and pandemic situations is crucial for several reasons. Social connections provide emotional support, reducing feelings of isolation, stress, and anxiety that often accompany such crises [36,37]. During disasters, strong social networks can facilitate the sharing of vital information, resources, and aid, enhancing community resilience and individual well-being. Additionally, connectedness ensures that vulnerable populations, such as the elderly or those with disabilities, receive the necessary care and support [37,38]. In the context of pandemics, where physical distancing measures are implemented, maintaining social connections through virtual means helps uphold mental health and prevents the adverse effects of loneliness [39]. Social connectedness also fosters a sense of community and solidarity, which is crucial for collective action and adherence to public health measures. Overall, maintaining social bonds during crises is vital for both psychological health and effective disaster response and recovery [25,40].

### Impact of Digital Technologies During the COVID-19 Pandemic

Digital health technologies are applicable in various areas, such as chronic disease self-management, as well as storing, sharing, and transferring confidential clinical data. These apps have enhanced the adherence to therapeutic regimes and provided a reliable platform for communication with health care professionals, enabling timely interventions [41-43]. Previous research on telemedicine has demonstrated its ability to save costs and travel time, particularly benefiting older populations in underserved areas. It can also reduce waiting times for patients for medical appointments, thereby reducing the time to diagnosis [43,44]. Additionally, especially during times of pandemics, it can help to reduce the risk of contagion and infections [45].

Most older individuals in the community relied on local health care workers and family members for food, water, and sanitary supplies, exposing themselves to unwanted health and safety risks. During this period, information about support services and preventive measures was disseminated to the public through digital technologies, including television, radio, health websites, and social media. However, the results of this study revealed that nearly half of the community people had limited knowledge of using this technology and were dependent on family members to attain important information. Furthermore, two-thirds of respondents reported challenges associated with internet connections, including high costs, limited accessibility, and difficulties using the internet. These issues are compounded by a gap in communication and information sharing, suggesting that digital technology is not equally accessible, resulting in a disparity in its use. These findings align with the report published by the Department of Internal Affairs in 2019, which stated that Māori people had limited access to internet connections and digital technologies, and inadequate funding for basic digital training [25]. These challenges highlighted the need for training and education to enhance the foundational digital skills for Māori communities [46].

There was a clear need for improved communication and a more effective system to reach COVID-19 victims. Information and information-sharing hubs could be better utilized to facilitate community links and support client work. Although the use of a QR scanning app enabled easy access to and management of information, a more effective approach would have been to maintain an up-to-date database with each community registered. The database would have helped people understand COVID-19 requirements and contributed to building the community [47,48]. Moreover, the inconsistency in the information shared by local health providers clearly indicated a lack of coordination among service providers [49,50]. This could have been further improved by exchanging and updating information in a timely manner and delivering one-on-one service as required [50,51].

### Impact of Digital Technologies During Cyclone Gabrielle

The frequent occurrences of extreme weather events have a significant impact on social connectedness within communities. Disasters can disrupt these social networks, displace individuals from their homes, and create feelings of isolation and loneliness. This is particularly important for elderly populations, who may rely heavily on their social connections for support and companionship. Recognizing and preserving social connectedness are crucial for effective disaster management and community resilience [52]. The findings of this study indicate that digital technology plays a key role in maintaining health and well-being during crises, as vital information can be shared through social connectedness. The lack of digital literacy and reliable telecommunications infrastructure disproportionately affected Māori communities’ ability to access timely information on safe practices during the COVID-19 pandemic and Cyclone Gabrielle, highlighting the urgent need for targeted interventions. Strengthening community-driven initiatives and integrating indigenous knowledge into disaster management plans can enhance resilience and support [53].

According to a recent report, the displacement triggered by Cyclone Gabrielle has destroyed numerous houses and forced a significant number of evacuations, affecting approximately 10,500 individuals. In the Opotiki district alone, heavy rainfall led to the evacuation of 1335 individuals, accounting for a substantial 15% of the total population. Despite these challenges, the affected communities have shown remarkable resilience [54]. Our study, a detailed analysis of the impact of Cyclone Gabrielle on digital public health, employed a qualitative research method. Like most studies of extreme weather events, we have reported that Cyclone Gabrielle had severe and long-lasting effects on public health. The individuals who faced this cyclone, coupled with power cuts and loss of internet connection, have experienced a significant impact on their psychological well-being and quality of life. This led to an increased level of anxiety, depression, and post-traumatic stress, which are likely to persist in the long term [55]. Within the affected region, the long-term devastation of primary industries, coupled with widespread damage such as heavy flooding, landslides, and infrastructure destruction, has necessitated a national state of emergency. Immediate action is crucial to mitigate the long-term effects of this disaster [56].

During such extreme weather events, poor access to reliable telecommunication services (like phones and internet connections) is exacerbated within populations that already face challenges such as remoteness [57]. The reliance on care support is a significant factor influencing an individual’s awareness of risks and ability to control them [58].

### Recommendations

It is essential to remember that Indigenous communities are incredibly diverse, each with its unique challenges and opportunities for digital inclusion. Addressing these challenges necessitates a multifaceted, collaborative, and, most importantly, a long-term commitment from governments, technology companies, nonprofits, and Indigenous communities. Each stakeholder plays a crucial role in developing sustainable and culturally appropriate solutions [3,59]. Therefore, based on the study findings and the considerations emerging from various literature and public reports, we suggest the following recommendations to help Indigenous communities overcome challenging situations, such as the pandemic and extreme weather events.

The need for a user-friendly and culturally relevant digital solution/technology was evident, as older individuals found it difficult to learn how to use digital technology. The frustration caused by the long waiting time for the national COVID 0800 number was accepted by those affected by the outbreak. A local COVID number could have replaced this to facilitate the needs of the local community and its people [50,60]. The need for a more user-friendly, culturally relevant, and simplified version of the digital solution is paramount, particularly for older individuals, to make it “fit for purpose” [25,61]. It is crucial that these programs are community-led, culturally safe, and accessible to people of all ages [3,62], especially to reduce digital disparity among Māori communities.

Ensure local funding—people directly linked to the local community were expected to have more access to food, water, and medical supplies [63]. There is sufficient evidence that the complex administrative requirements and logistical challenges associated with accessing government funding and programs can pose significant threats to Indigenous communities with limited resources [64]. More local funding needs to be provided for low-income families coping with isolation, food scarcity, and health conditions [60].

The need for additional community services**—**specifically, health care for older people—was needed. Further support (eg, doctor visits, regular phone calls to check up on health) for older individuals who would also have been considered [52], particularly helping them access the information and the health requirements [65], for example, how to download and use a mobile app [3]. The government needs to apply the lessons learned to those in most need and work with the local communities to provide additional services [50,60].

Establish a training program for the local community—focusing on digital apps such as online banking, social media, and email, as well as accessing information on the internet. Many community members, especially older adults, lack proficiency in these areas. By providing training and education, older adults will develop fundamental skills, enabling them to utilize technology for their future needs [56,66,67]. Government support is crucial in ensuring equitable access to digital technologies and resources, particularly for underserved communities and those lacking digital literacy. With the help of the relevant authority, necessary training and resources can be provided to help individuals develop the digital skills needed to participate more fully in the digital economy [68,69].

Fostering leadership and partnerships—governments and mainstream service providers may overlook the unique needs and priorities of diverse Indigenous communities, failing to tailor policies and programs to their specific contexts. The absence of Indigenous-led initiatives is particularly concerning, as they are not only crucial but also the most effective way to develop culturally relevant and effective digital inclusion strategies. Recognizing the expertise of Indigenous communities in understanding their own needs is essential for respectful and impactful policy-making [25,70]. The implementation of these strategies necessitates fostering collaboration between local health care providers, regional and government funding bodies, community health partners, and the national health service in New Zealand (Te Whatu Ora) [31].

### Strengths and Limitations of the Study

The Kaupapa Māori was chosen for several reasons: first, the primary investigator identifies as Māori and is connected to the community through whakapapa or genealogical ties and developed a body of knowledge to improve Māori health status over her academic career; second, the elders from her community approached her to investigate their concerns about the lack of access to health services within rural and remote communities in their tribal region; and third, this publication is a continuation of a 3-phase study that utilized Kaupapa Māori methodology since its commencement [48]. This study also followed Standards for Reporting Qualitative Research (SRQR), which improved the clarity, completeness, transparency, and credibility of the study [71] (Checklist 1).

To ensure cultural safety protocols and kaupapa Māori methodology were adhered to, 2 female kaumatua or elders (RS and JT) assisted with access to the various communities and remained present throughout the interviews. This approach ensured that the elders provided a moderating influence on the research process, and Māori cultural protocols were adhered to. Unexpectedly, Cyclone Gabrielle occurred partway through the interviews, prompting the research team to include accounts from participants about this event during the data collection phase. However, a caution has been raised regarding the structured interviews to ensure they cover both the COVID-19 pandemic and Cyclone Gabrielle equally. Moreover, limitations include the small number of participants and reliance on family members to assist with interviews, which may affect data accuracy.

Another limitation was that the exact age of the participants was not collected or documented. The precise ages of individuals—especially older people—sometimes could not be determined. The exact age could only be determined if the birth was recorded. Moreover, in traditional Māori and Pacific societies prior to European contact, written birth records were not kept. People often tracked age in relation to events (such as wars, voyages, famines, and natural disasters) rather than exact dates.

### Conclusions

Vulnerable situations such as pandemics and extreme weather events can have tremendous effects on the lives of Indigenous people who live remotely. This study provided insights into how the COVID-19 pandemic and Cyclone Gabrielle posed significant challenges for Māori communities, particularly for vulnerable older individuals, highlighting the importance of digital technology and telecommunication services in maintaining social connectedness and accessing health care services. The study also focused on the actions that should be taken to mitigate these challenges and overcome difficult circumstances, such as the pandemic and Cyclone Gabrielle. The recommendations include a better health care system and improved coordination among care providers, user-friendly digital solutions, ensuring local funding and community services, establishing training processes for basic digital skills, and fostering leadership and partnerships with Indigenous New Zealanders.

## Supplementary material

10.2196/73974 Checklist 1 SRQR checklist. This study followed Standards for Reporting Qualitative Research (SRQR) [71].
